# Fast Interrogation of Fiber Bragg Gratings with Electro-Optical Dual Optical Frequency Combs

**DOI:** 10.3390/s16122007

**Published:** 2016-11-26

**Authors:** Julio E. Posada-Roman, Jose A. Garcia-Souto, Dragos A. Poiana, Pablo Acedo

**Affiliations:** Department of Electronics Technology, Universidad Carlos III de Madrid, Av. Universidad 30, E-28911 Leganés, Madrid, Spain; jposada@ing.uc3m.es (J.E.P.-R.); dpoiana@ing.uc3m.es (D.A.P.); pag@ing.uc3m.es (P.A.)

**Keywords:** fiber Bragg gratings, dual optical frequency comb, sensor interrogation, dynamic measurements, ultrasounds

## Abstract

Optical frequency combs (OFC) generated by electro-optic modulation of continuous-wave lasers provide broadband coherent sources with high power per line and independent control of line spacing and the number of lines. In addition to their application in spectroscopy, they offer flexible and optimized sources for the interrogation of other sensors based on wavelength change or wavelength filtering, such as fiber Bragg grating (FBG) sensors. In this paper, a dual-OFC FBG interrogation system based on a single laser and two optical-phase modulators is presented. This architecture allows for the configuration of multimode optical source parameters such as the number of modes and their position within the reflected spectrum of the FBG. A direct read-out is obtained by mapping the optical spectrum onto the radio-frequency spectrum output of the dual-comb. This interrogation scheme is proposed for measuring fast phenomena such as vibrations and ultrasounds. Results are presented for dual-comb operation under optimized control. The optical modes are mapped onto detectable tones that are multiples of 0.5 MHz around a center radiofrequency tone (40 MHz). Measurements of ultrasounds (40 kHz and 120 kHz) are demonstrated with this sensing system. Ultrasounds induce dynamic strain onto the fiber, which generates changes in the reflected Bragg wavelength and, hence, modulates the amplitude of the OFC modes within the reflected spectrum. The amplitude modulation of two counterphase tones is detected to obtain a differential measurement proportional to the ultrasound signal.

## 1. Introduction

The good performance of fiber Bragg grating (FBG) sensors makes them useful in different environments and applications [1,2]. Examples of dynamic measurements using FBGs are monitoring ballistic tests on structures with pneumatic launchers or light gas guns, optical fiber sensing systems for structural health monitoring (SHM) of aircrafts and aerospace platforms, and the underneath of high-speed trains monitoring. In these kinds of applications, FBG arrays are embedded [3] or installed on the surface of materials structures to measure load, strain, temperature, vibration, acoustic emission, impact, and ultrasounds. Actual scenarios are those of a large deployment of sensors [4], of extreme conditions, and of fast dynamic measurements. FBG sensors can address two aspects of SHM at the same time: large range strain measurements (load monitoring) and fast detection of vibrations and ultrasounds (non-destructive techniques for damage detection) [5].

The interrogation of FBG arrays are based on a source that is common to all sensors in the array. It can be either a tunable laser source (TLS) or a broadband source (BBS). BBSs allow for a fast direct read-out, but the signal is attenuated at the spectral-analyzer (edge filter, interferometer, etc.). This low output signal problem is worse if the BBS is split between parallel arrays, or shared by wavelength multiplexed serial arrays. The TLS architectures optimize the output signal power, but the read-out frequency is limited by the repetition (tunability) rate.

On the other hand, optical frequency comb (OFC) generators are versatile systems that are well known for precision measurements of distance [6,7] and spectroscopy [8]. The electro-optic generation of OFC from a continuous-wave laser allows for more compact, robust and portable systems [9]. The use of OFC for FBG interrogation combines the advantages of broad-band and tunable laser interrogators as they interrogate the complete optical span with better power handling efficiency, which is critical in arrays.

Dual-optical frequency comb spectroscopy is a powerful technique that is currently being implemented in different instrumentation and measurement fields, as it allows for direct mapping of optical frequencies into radio frequency signals [10]. The incorporation of dual-optical frequency comb techniques would also allow for the detection of small changes in the spectrum of FBGs with a large dynamic range [11]. Electro-optic dual OFCs have been recently applied in spectroscopy to obtain more compact and portable systems for field applications [12], as they provide coherent broadband sources with higher power per line and independent control of line spacing and comb bandwidth. They offer flexible and optimized sources for the interrogation of other sensors, such as FBGs. 

In this paper, a dual-OFC is designed to operate within the reflected spectrum of a FBG and a direct read-out is obtained by mapping the optical spectrum onto the radio-frequency spectrum. A fast interrogation of one FBG within an array is obtained by simply detecting two radio-frequency tones, which are amplitude modulated by the optical fiber sensor. Measurements of vibrations and ultrasounds are demonstrated with this sensing system.

## 2. Dual-Optical Frequency Comb-Based FBG Interrogation System

### 2.1. The Interrogation Method

The new interrogation method proposed here take advantage of the dual-OFCs (DOFCs) to make smart use of the optical spectrum, exploiting its versatility and agile frequency configuration capabilities in order to enhance the sensitivity of a FBG sensor.

**Figure 1:**
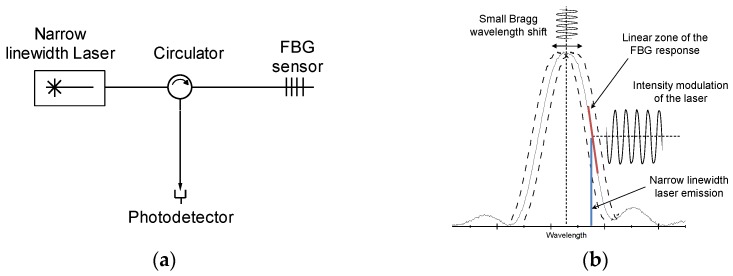
Interrogation of a fiber Bragg grating (FBG) sensor using a single mode laser. (**a**) Optical scheme of the interrogation system. (**b**) Example of the interrogation method.

An interrogation method commonly used, especially for measuring dynamic signals, is to illuminate the FBG in a linear region of the reflected spectrum using a single-mode narrow linewidth laser (Figure 1a); thereby, if there is a strain field on the FBG inducing a small shift of the Bragg wavelength (*λ_B_*), the intensity of the reflected laser mode will be observed with an amplitude modulation proportional to the shift of *λ_B_*, as is shown in detail in Figure 1b. Using this method with extremely narrow linewidth lasers (<100 kHz), a very high resolution was achieved in the order of 100 pε/√Hz [5,13].

**Figure 2:**
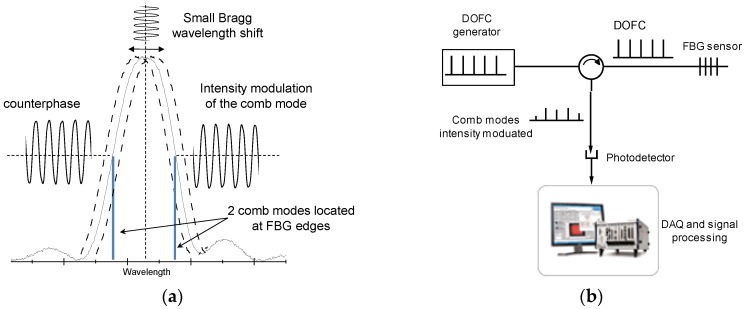
Proposed FBG interrogation system based on a dual optical frequency comb (DOFC). (**a**) Principle of the proposed interrogation method. (**b**) Simplified optical scheme of the proposed interrogation system.

The principle of the proposed interrogation system works in a similar way, by using two narrow linewidth modes of a DOFC to illuminate the two linear regions of a FBG, with the aim to obtain differential signals that are used to increase the sensitivity, as is shown in Figure 2a where the principle of the interrogation method is depicted. For simplicity, there is only one FBG in Figure 2; however, in principle, the multiple modes of a DOFC can be used for the interrogation of a serial array of FBGs. This can be done if a broad DOFC is generated for covering the optical spectrum of a FBG serial array. Currently, there is increasing interest in the expansion of OFCs for applications in several fields, and great research efforts are being focused on the generation of broader OFCs [14,15,16]. State-of-the-art electro-optical DOFCs are able to generate a broadband OFC of 40 nm [17]. A broadband DOFC like this might be used to interrogate a serial array with 13 FBG sensors spaced 3 nm apart. Moreover, FBG sensors in parallel network topologies could be also interrogated with the proposed interrogation approach.

Considering the schematic of the interrogation system shown in Figure 2b, which is illuminated by a DOFC, the comb modes that coincide within the spectrum of the FBG will be reflected and detected at the output. Assuming the reflected spectrum of each FBG in a serial array is a Gaussian function, the intensity of each comb mode at the detector can be written as

(1) $$I_{out}=\frac{1}{4}R\;P_{0}\;e^{\left[-4\;\ln(2)\;\cdot \;\left(\frac{\Delta \lambda }{\Delta \lambda_{B}}\right)\right]}$$

where *R* is the FBG maximum reflectivity (0 ≤ *R* ≤ 1), *P*_0_ is the optical power of the comb mode being reflected by the FBG, Δ*λ_B_* is the grating spectral width at half maximum, and Δ*λ* = *λ* − *λ_B_* is the difference between the Bragg wavelength of the FGB and the comb mode wavelength (*λ*). Differentiating Equation (1) in order to locate the maximum sensitivity zones, in which the two modes of the comb must be placed for best performance, it becomes

(2) $$\frac{\partial I_{out}}{\partial \Delta \lambda }=-2\;R\;P_{0}\ln(2)\frac{\Delta \lambda }{\Delta {\lambda_{B}}^{2}}\;e^{\left[-4\;\ln(2)\;\cdot \;{\left(\frac{\Delta \lambda }{\Delta \lambda_{B}}\right)}^{2}\right]}$$

**Figure 3:**
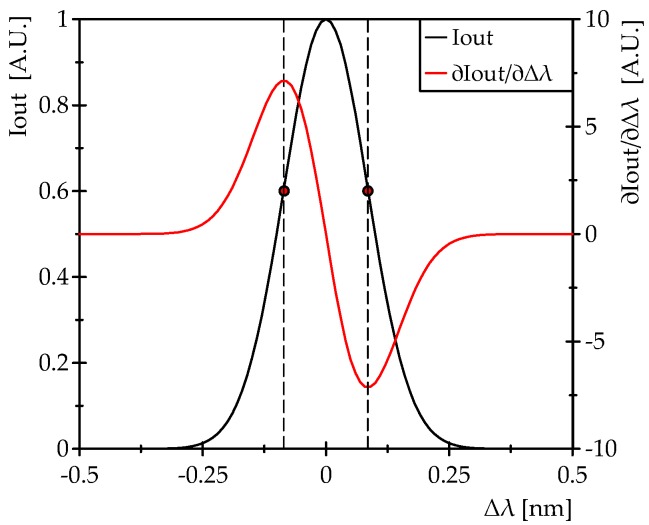
Intensity of each comb mode (*I_out_*) observed at the detector and the sensitivity (∂*I_out_*/∂Δ*λ*) as a function of Δ*λ*.

Figure 3 shows the intensity (*I_out_*) of each mode at the detector and the sensitivity (∂*I_out_*/∂Δ*λ*) as a function of Δ*λ*. It can be seen clearly that the two peaks in ∂*I_out_*/∂Δ*λ* are opposite in sign; therefore, if two comb modes are located in these points (marked in Figure 3), two counterphase signals (differential signals) are obtained whether the comb modes can be recovered without a mix between them, as is the case with the DOFC technique, which is explained below in Section 2.2.

### 2.2. Dual Optical Frequency Comb Generation

By means of the DOFC technique, it is possible to recover the comb modes reflected from a serial array of FBGs unambiguously. This is possible because, through the DOFC approach, the optical frequencies of comb modes are downshifted to a lower frequency range, in the detection bandwidth of a photodetector; hence, the optical comb is mapped into the radio-frequency (RF) spectrum [12].

**Figure 4:**
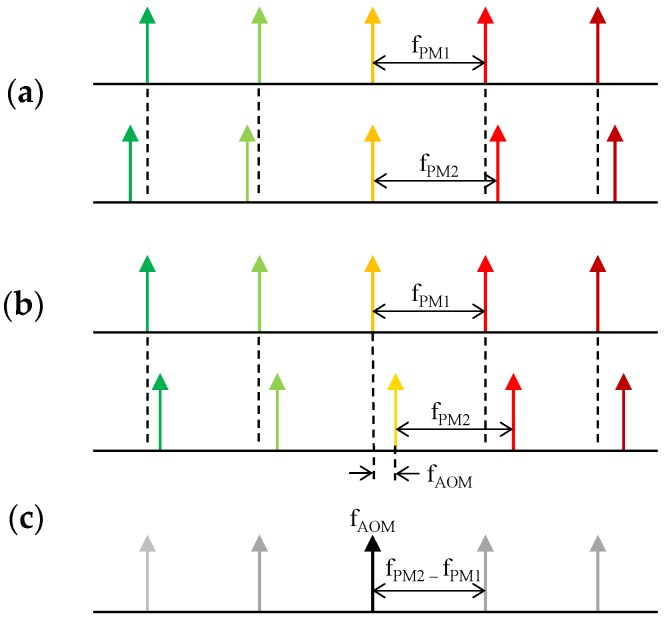
The DOFC approach. (**a**) A different frequency spacing is introduced to the combs and then (**b**) the central optical frequency of one of them is shifted at a frequency f_AOM_ using an acousto-optic modulator (AOM). (**c**) Resultant radio-frequency (RF) comb detected by a photodetector is centered at f_AOM_ and with a frequency spacing of f_PM2_ − f_PM1_.

A DOFC is formed by two OFCs with slightly different frequency spacings, as is shown in Figure 4a. Then, a shift in the optical frequency of one of them is introduced, which is done typically using an acousto-optic modulator (AOM); this is detailed in Figure 4b. When these two optical combs heterodyne on the surface of a photodetector, the resultant beat notes form an exact image of the optical comb but mapped into the RF band. The resultant RF comb, shown in Figure 4c, is centered at the shifting frequency introduced by the AOM (f_AOM_) and has a frequency spacing of f_PM2_ − f_PM1_, where f_PM1_ is the frequency spacing of the first comb and f_PM2_ the frequency spacing of the second comb. The generation of the two combs (Figure 4a), which are necessary to implement a DOFC, is usually done through the electro-optic phase modulation. In this case, the sidebands generated by the phase modulation become the optical comb.

Using the dual comb approach in the FBG interrogation system proposed here (Figure 2), any amplitude changes in optical comb modes reflected by the FBG will be observed in the RF comb (Figure 4c) with the same magnitude. Therefore, the comb modes located in the points of maximum sensitivity (Figure 3) become two amplitude modulated RF carriers once they are downshifted to the RF band. Thus, an AM demodulator can be used to recover these amplitude encoded measurements.

### 2.3. Implementation of the Interrogation System

**Figure 5:**
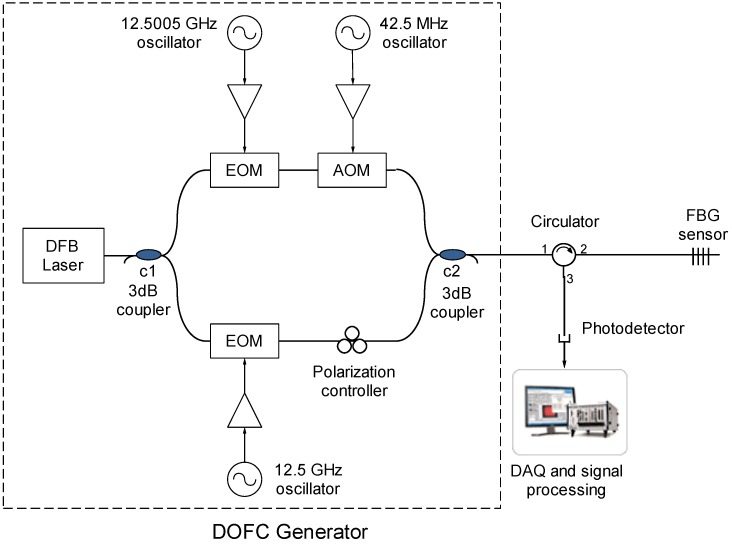
Scheme of the interrogation system.

**Figure 6:**
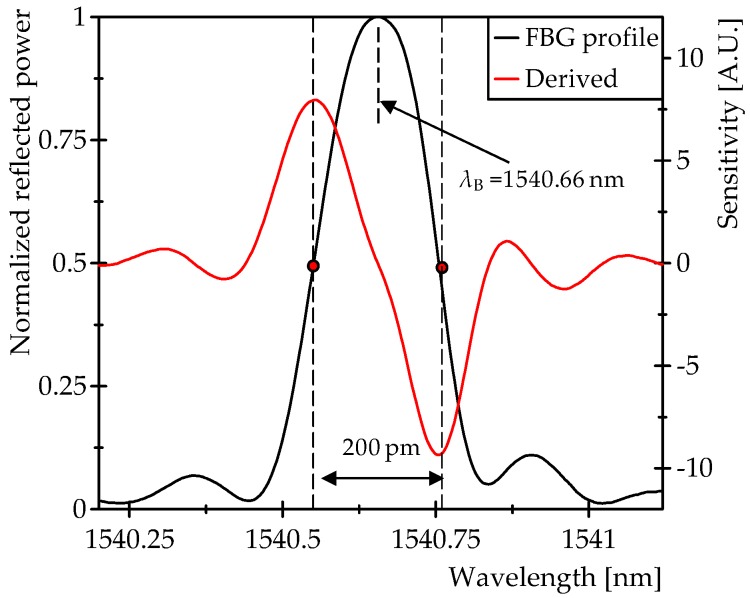
Characterization of the FBG sensor spectrum profile in reflection.

The scheme of the proposed interrogation system is shown in Figure 5. The first stage of the system is the DOFC generator. The light source used to illuminate the fiber system is a DFB laser with a spectral linewidth (FWHM) of 10 MHz (model: QDFBLD-1550-20, QPhotonics, Ann Arbor, MI, USA). Two electro-optic phase modulators (EOM) are used to generate the two OFCs (Figure 4a), each with a slightly different spacing among its modes. This frequency difference (f_PM2_ − f_PM1_) was selected to be 0.5 MHz in order to obtain enough bandwidth between the RF comb modes for the measurements of ultrasound signals in the experimental tests. On the other hand, f_PM1_ and f_PM2_ were determined in a previous characterization of the FBG that was performed with the aim of obtaining the maximum sensitivity points. In this characterization (Figure 6), the spectrum of a FBG in reflection was measured using an optical spectrum analyzer with a 20 pm resolution. The collected data were used to differentiate the reflected profile in order to find the maximum sensitivity points; this result is also shown in Figure 6. It was found that these points are spaced 200 pm, which is equivalent to ~25 GHz around the *λ_B_* = 1540.66 nm of the FBG. This means that, if the mode of the DFB laser source (central mode of the comb) matches the *λ_B_*, and the EOMs are modulated at frequencies near 12.5 GHz, then the two lateral modes adjacent to the central mode of the comb are tuned to the desired points. Therefore, f_PM1_ and f_PM2_ were selected to be 12.5 GHz and 12.5005 GHz, respectively. The signal of one comb is then shifted by an AOM driven at f_AOM_ = 42.5 MHz. These two OFCs are combined in a 3 dB coupler (c2) and finally come to a circulator, where Port 2 is connected to the FBG sensor and Port 3 is connected to a photodetector. All oscillators in the DOFC generator and the DAQ are clock synchronized.

The linear range of the FBG reflection spectrum profile is ±40 pm (Figure 6). Considering the strain sensitivity of a FBG (d*λ_B_*/dε = 1.2 pm/με @ 1.55 μm), the linear sensing range is ±33 με.

**Figure 7:**
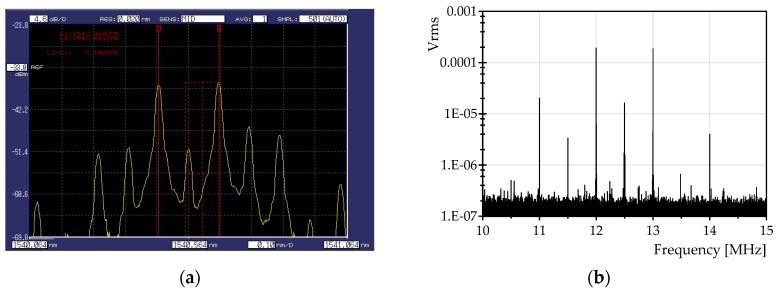
(**a**) Example of the comb measured at Port 2 of the circulator. (**b**) Reflected RF spectrum observed after the downsampling.

The detection is performed with a 150 MHz bandwidth InGaAs photodetector, and a band-pass filter is used to condition the RF signal. A downsampling of the RF comb centered at 42.5 MHz is performed digitizing it at a sampling frequency of 30 MHz, using a 12-bit resolution DAQ system (PXI-5105, National Instruments, Austin, TX, USA). Consequently, the digitized RF comb is observed centered at 12.5 MHz. An example of the DOFC measured at Port 2 of the circulator is shown in Figure 7a, and the corresponding RF comb observed after the digitization and downsampling is shown in Figure 7b.

**Figure 8:**
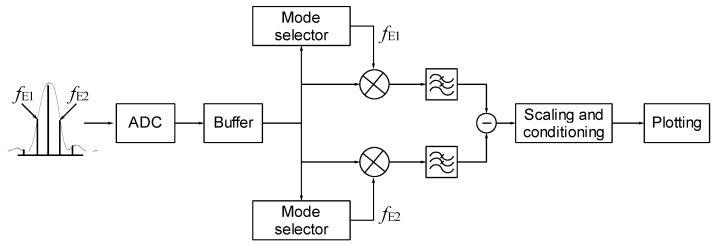
Block diagram of the digital AM demodulator.

Once the RF comb is digitized and downsampled, a digital AM demodulator implemented in LabVIEW is used to recover the measurement from the amplitude modulated comb modes. The simplified block diagram of the demodulator is shown in Figure 8.

In the AM demodulator, an intermediate buffer temporarily stores the data of the digitized RF comb. The two synchronized tones *f*_E1_ and *f*_E2_ are obtained through two mode selectors. Then, these tones are mixed with the digitized RF comb in order to shift the desired modes to baseband. The other comb modes are removed by the low pass filters and the differential signal is obtained subtracting both outputs. Finally, conditioning and scaling are performed in the subsequent stages.

## 3. Experimental Results

### 3.1. Experimental Set-up

**Figure 9:**
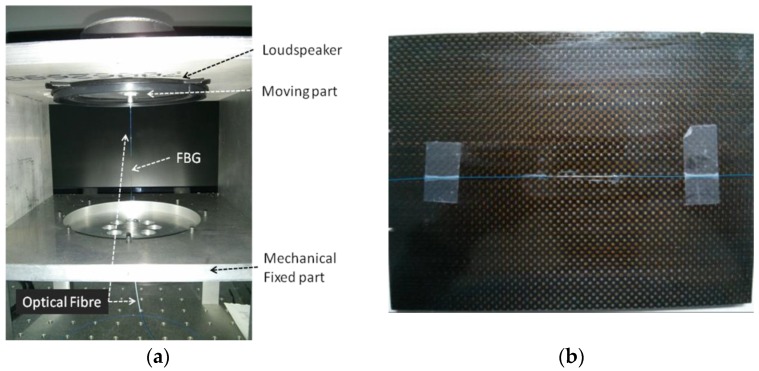
Experimental set-ups. (**a**) Test bench for measurement of vibrations. (**b**) Set-up for the detection of ultrasound on a composite plate.

For the proof of concept of this system, we prepared two experimental set-ups with the aim to measure two types of signals: vibrations and ultrasound signals. Both set-ups were implemented for the measurement using only one FBG sensor. For the excitation of the vibrations signals, the test bench shown in Figure 9a was implemented. It was built bonding a FBG sensor at one extreme to the surface of a loudspeaker that is used as actuator to induce dynamic strain onto the fiber. The other extreme of the fiber is bonded to a static surface that can be adjusted with screws in order to pre-stress the FBG. The length between the two bonded extremes of the fiber is 10 cm. With this set-up, it is possible to induce a dynamic strain of high amplitude with frequencies up to 2 kHz.

On the other hand, the set-up prepared for the measurement of ultrasound was implemented bonding an FBG with cyanoacrylate to the surface of a 7 mm thickness carbon fiber plate with dimensions of 15 cm × 21 cm. The bonding length of the fiber on the carbon fiber plate is 4 cm. In this set-up, the excitation of the ultrasound signals is done using different ultrasound transducers with resonance frequencies of 40 kHz and 120 kHz. A detail of this set up is shown in Figure 9b.

### 3.2. Measurement of Vibrations

**Figure 10:**
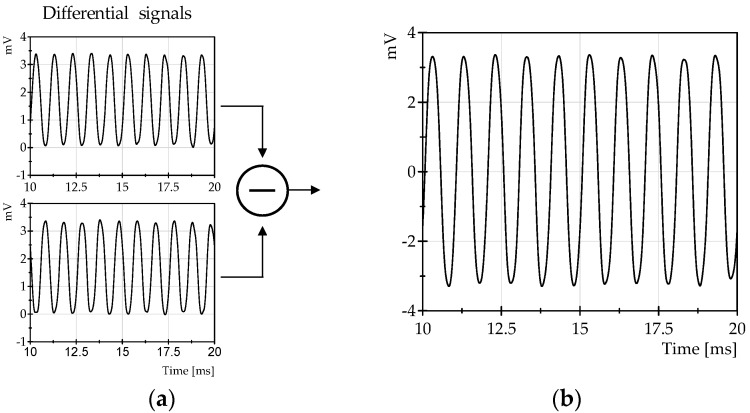
(**a**) Differential signals corresponding to a vibration of 1 kHz frequency. (**b**) Resultant signal corresponding to the subtraction of the differential signals.

The first test performed was focused on the measurement of vibrations in the range of 100 Hz–1 kHz, to test the system with this type of signal. This experiment was carried out in the test bench shown before in Figure 9a. The loudspeaker was excited at several frequencies in the aforementioned frequency range, and different amplitudes of vibration were applied. An example of the typical signal observed during a test with vibrations of 1 kHz is shown in Figure 10.

Figure 10a shows the corresponding differential signals recovered from the two optical comb modes located at the edges of the FBG, and Figure 10b shows the result after the subtraction of these differential signals. It can be observed that the amplitude, and hence the sensitivity, is approximately twice that obtained with one of the differential signals of Figure 10a. This is an interesting characteristic because, compared with other interrogation approaches, the sensitivity is increased without amplifiers; at the same time, common mode rejection is provided. The measured strain corresponding to the vibration signals was 56 με pk-pk.

**Figure 11:**
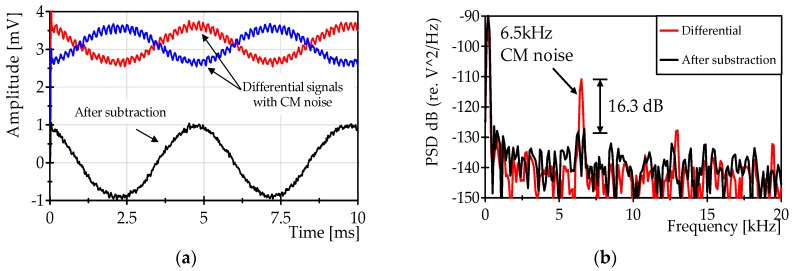
Measurement of common mode rejection: (**a**) Typical signals observed for a 200 Hz vibration when a 6.5 kHz common mode signal is added. (**b**) A spectrum of the signals is shown in (**a**).

On the other hand, an additional test was carried out in order to evaluate the common mode rejection that could be provided by the differential detection. In this test, a common mode signal of 6.5 kHz was introduced through an intensity modulation of the light illuminating the fiber system, while the vibration test bench was excited with a frequency of 200 Hz. The differential signals with the added common mode noise are shown in Figure 11a, together with the resultant signal after the subtraction. Figure 11b show the power spectrum of the signals of Figure 11a.

The magnitude of the 200 Hz vibration signal corresponds to 18 με measured with the FBG (Figure 11a). The results of the test reveal that there is an attenuation of approximately 16 dB for the common mode signals. This characteristic could be useful to minimize the effects of common mode noises such as relative intensity noise of the laser source, which strongly affects the performance of a fast FBG interrogation system.

### 3.3. Measurement of Ultrasounds

One of the objectives of this research work is the application of the proposed FBG interrogation system to the measurement of ultrasounds. Fast interrogation of dynamic signals such as ultrasounds with FBG sensors is a challenge because these are detected in the range of nano-strain and even in the pico-strain range. Therefore, a high resolution is needed for the detection of such signals.

**Figure 12:**
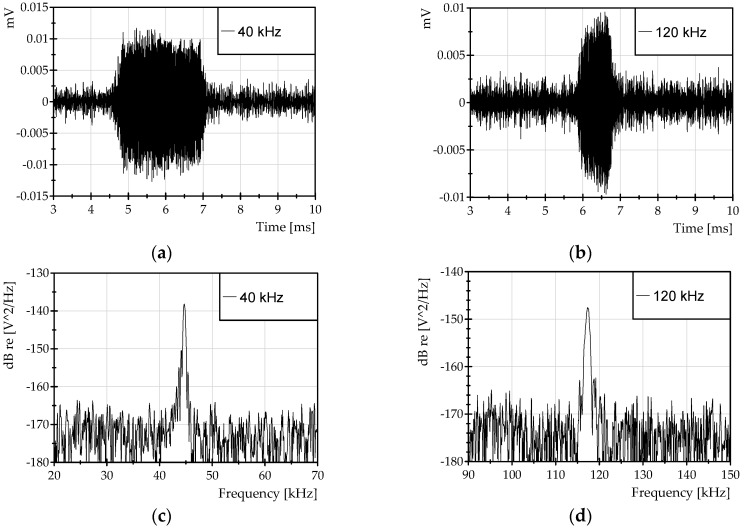
Experimental results of ultrasound measurements: detected burst signals of (**a**) 40 kHz and (**b**) 120 kHz; power spectral density of the detected burst signals of (**c**) 40 kHz and (**d**) 120 kHz.

The experiments with ultrasound signals were carried out with a FBG bonded to a carbon fiber plate (Figure 9b). The types of signals excited during the test were bursts with frequencies of 40 kHz and 120 kHz. In both cases, the burst duration was 100 cycles of the fundamental frequency. The typical signals observed during the tests are presented in Figure 12a,b. The corresponding power spectral density of the signals in Figure 12a,b is shown in Figure 12c,d, respectively.

The ultrasound signals were detected with amplitudes of 0.4 με for the case of the 40 kHz burst, and 0.32 με for the 120 kHz burst. The minimum detectable amplitude of the burst signal that can be distinguished in Figure 12a,b corresponds to ~50 nε (a detection bandwidth of 50 kHz).

## 4. Conclusions

DOFCs have been proposed for the fast interrogation of FBGs. A dual-comb is designed to operate within the reflected spectrum of the FBG and a direct read-out is obtained by mapping the optical spectrum onto the RF spectrum. A fast interrogation of each FBG in an array is obtained by simply detecting RF tones around 42.5 MHz with a spacing of 0.5 MHz.

The system is able to detect and process signals of vibrations and ultrasounds in real time. It was tested with dynamic strain vibrations (200 Hz and 1 kHz, 18 με and 56 με) and ultrasounds (40 kHz and 120 kHz, 0.4 με and 0.32 με). Moreover, the differential detection provided by the interrogation system enables a rejection to common mode noise. Sixteen decibels of common mode attenuation was observed in the experimental tests. The minimum detectable ultrasound amplitude is 50 nε, limited by the observed background noise. The experiments were carried out in set-ups with one FBG sensor; however, it is possible to interrogate serial or parallel arrays of multiple FBG sensors with different *λ_B_* due to the spectral characteristics of the DOFCs.

The Bragg wavelength may drift due to temperature and static strain. To overcome this, a wavelength tracking mechanism based on a tunable laser source is proposed (e.g., using a VCSEL in the DOFC [9]). Moreover, electro-optical OFC generators are reconfigurable in terms of their main parameters—the frequency spacing of the comb modes, the spectral width (number of comb modes), and the central wavelength—which are adjustable independently. Therefore, the DOFC can be tuned to optimize the response of the sensing system to vibrations or ultrasounds specifically.
